# Metabolic Stress in the Immune Function of T Cells, Macrophages and Dendritic Cells

**DOI:** 10.3390/cells7070068

**Published:** 2018-06-29

**Authors:** Charlotte Domblides, Lydia Lartigue, Benjamin Faustin

**Affiliations:** 1 Bordeaux University, ImmunoConcept, 33000 Bordeaux, France; charlotte.domblides@chu-bordeaux.fr; 2 CNRS, UMR 5164, 33000 Bordeaux, France; 3 Department of Medical Oncology, Hôpital Saint-André, Bordeaux University Hospital-CHU, 33000 Bordeaux, France; 4 INSERM U1218, Bergonié Institute, Bordeaux University, 33000 Bordeaux, France; lydia.lartigue@gmail.com; 5 Takeda California, Inc., 10410 Science Center Drive, San Diego, CA 92121, USA

**Keywords:** Immunology, metabolism, innate immunity, adaptive immunity, metabolic stress

## Abstract

Innate and adaptive immune cells from myeloid and lymphoid lineages resolve host infection or cell stress by mounting an appropriate and durable immune response. Upon sensing of cellular insults, immune cells become activated and undergo rapid and efficient functional changes to unleash biosynthesis of macromolecules, proliferation, survival, and trafficking; unprecedented events among other mammalian cells within the host. These changes must become operational within restricted timing to rapidly control the insult and to avoid tissue damage and pathogen spread. Such changes occur at high energy cost. Recent advances have established that plasticity of immune functions occurs in distinct metabolic stress features. Evidence has accumulated to indicate that specific metabolic signatures dictate appropriate immune functions in both innate and adaptive immunity. Importantly, recent studies have shed light on whether successfully manipulating particular metabolic targets is sufficient to modulate immune function and polarization, thereby offering strong therapeutic potential for various common immune-mediated diseases, including inflammation and autoimmune-associated diseases and cancer. In this review, we detail how cellular metabolism controls immune function and phenotype within T cells and macrophages particularly, and the distinct molecular metabolic programming and targets instrumental to engage this regulation.

## 1. Introduction

Immune cells arise from a common pluripotent hematopoietic stem cell that upon differentiation produces two main lineages, referred as the myeloid and lymphoid lineages. While the former fully maturate in the bone marrow and generate components of the innate immune system, the latter generally reach lymphoid organs (i.e., such as the thymus) to complete maturation and produces most cellular constituents of the adaptive immune system. Once functional, these immune cells undergo several modifications of their genetic programming in response to an encounter, either in sterile or non-sterile conditions. These genetic modifications lead to the transition between quiescent, activated, and memory states, and control immune polarization. At the intracellular level, these transitions are accompanied by major metabolic changes. Cells notably modulate bioenergetic requirements, building blocks for their proliferation and survival (nucleotide, lipids, amino acids, and protein synthesis), and oxidative stress. Transcriptional and post-transcriptional regulation of these genes plays a key role in the function of immune cells, dependently on the availability of various nutrients and presence of oxygen. The immune response is associated with dramatic modifications in tissue metabolism, including depletion of nutrients, increased oxygen consumption, and the generation of reactive nitrogen and oxygen intermediates. These modifications are in part due to the recruitment of various inflammatory cells.

**Figure 1:**
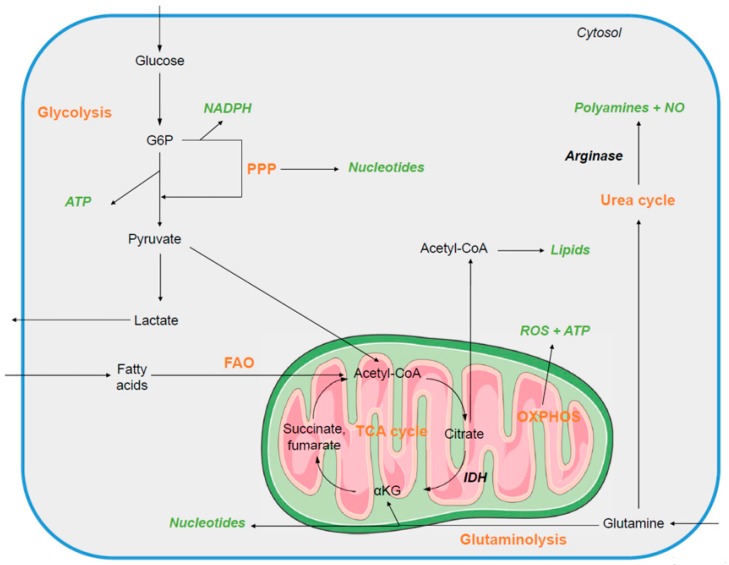
The primary metabolic pathways in quiescent immune cells. The main catabolic pathways (orange) that contribute to the production of macromolecules or metabolites (green) in quiescent mammalian immune cells are the mitochondrial tricarboxylic acid (TCA) cycle and oxidative phosphorylation (OXPHOS) for ATP synthesis, fatty-acid oxidation (FAO), glutaminolysis, and urea cycle.

Glucose is a well-known nutrient for energy supply that feeds glycolysis and is converted into pyruvate in the cytosol (Figure 1). Under normoxic conditions, pyruvate is transformed into acetyl-Coenzyme A (CoA) in the mitochondria by oxidative decarboxylation. The oxidative phosphorylation system (OXPHOS) is the most efficient way for mammalian cells to produce energy, in contrast to the other Adenosine TriPhosphate (ATP)-producing pathway, glycolysis [1]. Mammalian cells can metabolize other substrates for energy supply depending on the tissue or physiological contexts, such as fatty-acids (through mitochondrial β-oxidation) or glutamine (via glutaminolysis). Glutamine is the most abundant circulating free amino acid. In normal physiological conditions, glutamine is converted into α-Ketoglutarate to feed and sustain the mitochondrial tricarboxylic acid (TCA) cycle. However, some cancer cells upregulate a metabolic pathway that transforms α-Ketoglutarate into acetyl-CoA to support fatty acid biosynthesis and sustain tumor cell proliferation [2].

Another cellular method of producing ATP is glycolysis-mediated fermentation. Under hypoxic conditions, pyruvate remains in the cytoplasm and is mostly converted into lactate rather than acetyl-CoA. This reaction is catalyzed by lactate dehydrogenase (LDH). This glycolytic pathway is more rapid but less efficient than OXPHOS for ATP synthesis. Fermentation under normoxic conditions is aerobic glycolysis. The metabolic switch from mitochondrial oxidative to aerobic glycolysis has been observed in cancer cells from solid tumors and is known as the Warburg effect [3]. This shift enables cancer cells to cope with strong environmental constraints, including hypoxia, and therefore sustain rapid generation of precursors essential for high proliferation and survival. High rates of glycolysis, glutaminolysis, and epigenetic reprogramming allow for the use of glycolytic-branched pathways, denoted as salvage pathways, that lead to nucleotide, lipid, and amino acid synthesis and detoxification [4]. Importantly, glutaminolysis uses cancer-specific enzyme isoforms that allow for citrate production, which improves the rate of lipid synthesis. Mitochondrial energy metabolism is mostly down-regulated with a broken TCA cycle that accumulates intermediates, including succinate and fumarate. These TCA cycle-derived metabolites regulate epigenetic reprogramming and recently have been referred to as oncometabolites [5]. Finally, some cancer cells strongly upregulate the glycolytic-branched pentose phosphate pathway (PPP) to sustain fatty acid biosynthesis through Nicotinamide Adenine Dinucleotide Phosphate (NADPH) production [6]. Recently, more attention has been given to the role of this metabolic pathway in cancer phenotype, because of its multiple roles in nucleotide synthesis and energy production, thereby sustaining tumor cell proliferation and survival. Furthermore, the PPP pathway is involved in the reduction of oxidative stress undergoing in cancer cells.

Similar to cancer cells, immune cells modify their metabolic requirements to acquire appropriate immune function in response to infection or tissue stress. Such metabolic modifications allow them to mobilize all the resources required to quickly proliferate and/or get fully activated to deploy their host-defense mechanisms. In lymphoid cells, for instance, going from a naïve/quiescent to a fully active immune effector cells implies a switch from an OXPHOS-dependent catabolic condition to a highly glycolytic anabolic state. After hazard clearing, a reversed metabolic shift then should occur in the remaining memory cells, reacquiring an oxidative non-proliferating state. As for the myeloid component, metabolic changes occur upon M1 or M2 macrophages polarization, towards higher (i) glycolytic flux and (ii) OXPHOS and fatty acid oxidation (FAO), respectively. At last, dendritic cells also switch to high glycolysis upon activation for antigen presentation. All these changes rely on complex metabolic networks that comprise numerous signaling pathways and key metabolic mediators/actors, described now to specifically control immune cellular functions at various levels. The metabolic peculiarities of various immune cell subtypes that drive their immune functions in normal and diseased conditions, including inflammatory diseases or during the antitumor immune response, are discussed below.

## 2. Metabolic Networks in Lymphoid Cells

Most lymphoid cells (T and B cells) are crucial components of the adaptive immune system that provide specific recognition of “non-self” [7], as well as “disturbed-self” under some circumstances, including cancer cells through the carcinogenesis-induced expression of neoantigens. Natural killer (NK) cells belong to the subtype of lymphoid cells managing primarily cytotoxic innate immune responses. B cells maturate in the bone marrow and T cells in the thymus. Lymphocytes then enter the circulation and peripheral lymphoid organs where they survey for pathogens or tumor cells. Upon exposure to antigens, these lymphocytes first differentiate into the effector phenotype and subsequently the memory phenotype. B cells can eradicate pathogens by releasing antibodies and effector T cells can produce cytotoxic granules or signal to other immune cells to coordinate the adaptive immune response. A small proportion of these activated effector T cells does not undergo cell death and become memory T cells that remain in the peripheral lymphoid organs and remain potent to antigen recalls.

### 2.1. Function and Activation of T Cells

**Figure 2:**
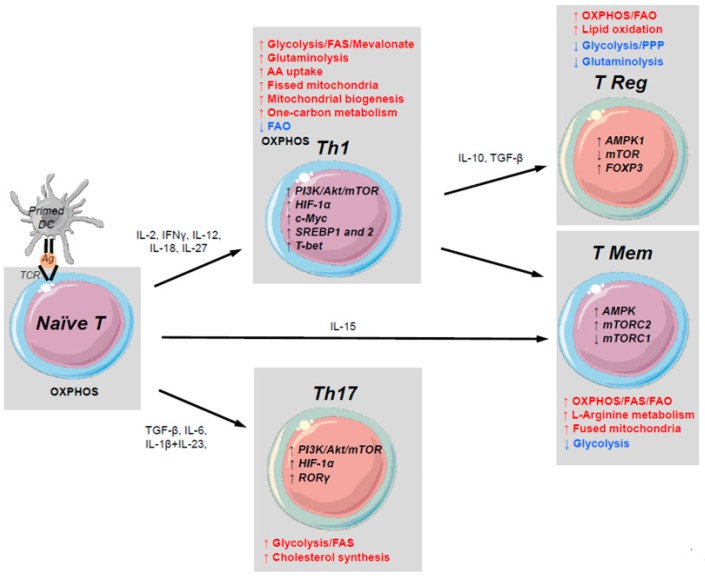
Metabolic rewiring following T cell activation. Metabolic requirements are indicated for the differentiation of quiescent naive T cells into Th1, Th17, TRegs, or T memory immune phenotypes. The primary metabolic pathways for each immune phenotype that are upregulated, downregulated, or unchanged are indicated in red, blue, or black respectively. The primary metabolic markers involved in these pathways within immune cells are indicated in bold black (metabolic enzymes or transcription factors). Soluble immune markers that regulate these T cell phenotypes are indicated in black (cytokines, growth factors). PI3K, phosphoinositide 3-kinase; Akt, protein kinase B; mTOR, mammalian target of rapamycin; HIF-1α, hypoxia-inducible factor 1-alpha; AMPK, AMP-activated protein kinase; SREBP, sterol regulatory element-binding protein; T-bet, T-box expressed in T cells; FAS, fatty-acid synthesis; AA, amino acids; FAO, fatty-acid oxidation; RORγ, RAR-related orphan receptor gamma; FOXP3, forkhead box P3; PPP, pentose phosphate pathway; TCR, T cell receptor; Ag, antigen.

T lymphocytes are sentinel cells that trigger an antigen-specific response. They play a crucial role in controlling infection and the pathogenesis of numerous human diseases in sterile conditions, including inflammatory and autoimmune diseases and cancer. Naive T cells recognize antigens as processed peptides presented by the major histocompatibility complex (MHC) molecular machinery that is expressed by antigen presenting cells (APCs). This interaction leads to T cell activation, rapid proliferation, and production of various effector molecules that enable the control of disease-causing pathogens. After interaction with the antigen, most effector T cells die through activation-induced cell death (AICD), leaving only a small number of activated T cells to become memory T cells. Memory T cells stably persist and can rapidly and more potently respond to antigen recalls. Each of these phenotypic transitions require a fine-tuning of metabolic networks that are necessary to cope with environmental constraints (e.g., hypoxia, nutrient deprivation), and to acquire novel immune functions. Recent progress has been made to better define the various underlying transcriptional metabolic programming, and to better understand how this programming controls immune signaling pathways and ultimately T cell function (as shown in Figure 2).

### 2.2. The Metabolism of Naive/Quiescent T Cells

During their quiescence in a limited proliferative state, T cells display catabolic metabolism in which nutrients are used for homeostasis and maintenance but not for biosynthesis (Figure 2). Thus, naïve T cells use glucose, lipids and amino acids to supply OXPHOS [8,9,10] in order to maintain housekeeping functions. These cells have low rates of glycolysis and glutaminolysis. This quiescent phenotype is maintained via extracellular signaling (such as Interleukin-4 (IL-4) and IL-7 cytokines) [11,12]. T cells cultured without these cytokines die because of a bioenergetic decline and reduced expression of Bcl-2 [13]. Quiescence is maintained through the inhibition of the protein kinase B (Akt) pathway and T Cell Receptor (TCR) signals by environmental sensors. Tuberous sclerosis 1 (TSC-1) is a Akt pathway inhibitors and its deletion leads to a premature exit from quiescence [14], indicating that TSC-1 dependent control of Mammalian Target Of Rapamycin (mTOR) is crucial in maintaining the quiescence of naive T cells.

### 2.3. Metabolic Switch in Effector T Cells and Its Regulation

#### 2.3.1. Metabolic Reprogramming

Upon TCR-dependent recognition of antigens, downstream metabolic reprogramming leads to rapid growth, proliferation, and acquisition of specific T cell functions, by providing a boost of energy supply and a molecular framework suitable for the induction of immune signaling pathways. This metabolic reprogramming results in a shift toward aerobic glycolysis with an upregulation of the PPP, glutaminolysis to produce ATP, and an increase in biomass such as amino acids, lipids, and nucleic acids to provide necessary cellular building blocks [15]. Thus, effector T cells shift to an anabolic metabolic programming to incorporate nutrients into biomass for sustaining rapid proliferation. Therefore, effector T cell metabolism is comparable to some solid tumor cells that engage a Warburg-like metabolic rewiring. Even in the presence of oxygen, antigen-experienced lymphocytes become highly dependent on aerobic glycolysis for ATP synthesis, with an increased production of lactate coming from the upregulation of LDH-A gene expression and a strong rise of the glycolytic rate [16,17,18]. Such a process is Hypoxia-Inducible Factor (HIF)-1α dependent [19,20] and controls the over-expression of glucose transporter-1 (GLUT-1) [21]; deletion of GLUT-1 impairs T cell activation and function [22,23,24,25].

Activated T cells maintain mitochondrial OXPHOS and can use OXPHOS or aerobic glycolysis, dependent on environmental nutrient deprivation, although, aerobic glycolysis appears to be necessary for optimal cytokine production [26,27] (Figure 2). Consistent with the role of mitochondria, a recent study focusing on the temporal dynamics of T cell activation in vitro by using proteome and phosphoproteome profiling has shown that TCR activates tightly connected functional gene modules, kinases, and transcription factors. In this network, TCR-induced mTOR Complex 1 (mTORC1) leads to mitoribosome biogenesis and mitochondrial Complex IV activity, where COX10 subunit has a crucial role in the T cell exit from quiescence [28]. During glucose deprivation, glutamine can be used as an alternative pathway for energy supply [23,29]. Glutaminolysis is therefore essential for T cell activation and function [30,31], and glutamine depletion blocks proliferation and cytokine production. Furthermore, T cell activation is associated with increased levels of glutamine in a CD28-dependent manner that is linked to an increased expression of glutamine transporters. Their deletion leads to an impaired transition to the effector T cell phenotype [23,25]. These features have been associated with Extracellular signal–Regulated Kinases (ERK) function, thereby providing a molecular link to TCR signaling.

Another important metabolic component allowing T cell effector differentiation and expansion is the upregulation of fatty-acid synthesis, notably through the role of acetyl coenzyme A carboxylase (ACC1) essential for the conversion of acetyl coenzyme A to malonyl coenzyme A [32].

#### 2.3.2. Regulators of the Metabolic Switch in Effector T Cells

Regulation of this metabolic switch occurs at two different levels through transcriptional programming (involving c-Myc and estrogen-related receptors [ERRs]) and post-transcriptional programming (that involves AMPK activation and HIF-1α stabilization) [33]. Expression of c-Myc has several roles; it promotes glycolytic programming and supports aerobic glycolysis and it upregulates glutaminolysis in order to generate α-ketoglutarate to feed the mitochondrial TCA cycle. Furthermore, c-Myc favors glucose-derived citrate production for its use by fatty-acid synthesis [29]. Deletion of c-Myc in mouse models leads to the inhibition of TCR-induced glycolysis and glutaminolysis in T cells. These properties of c-Myc are similar to those in cancer cells [34]. ERRs are also important for the regulation of metabolic pathways [35]. ERRα regulates metabolic pathways that are important for T cell activation, differentiation, and effector functions, and ERRα levels increase during these processes. A ERRα-deficient mouse model showed that loss of expression leads to the suppression of GLUT-1 upregulation, glucose uptake, and mitochondrial processes, thereby blocking T cell growth and proliferation [35].

mTOR is a master regulator of T cell metabolism, integrating signals provided by nutrient levels and energy in T cells, cell stress pathways, and TCR and growth factor signals. CD28-dependent signaling involves the PI3K/Akt/mTOR pathway to promote glucose metabolism and to prevent T cell anergy [36]. mTOR has 2 isoforms: mTORC1 and mTORC2 [37]. The activation of PhosphoInositide 3-Kinase (PI3K) leads to the recruitment of Akt isoforms 1 to 3 and 3-phosphoinositide-dependent protein (PDPK1) to the cell membrane. PDPK1 can thus phosphorylate and activate Akt, and this cascade leads to the activation of mTORC1 [38]. Akt and mTORC1 promote GLUT-1 translocation to the cell surface, prevent its internalisation, lead to the expression of amino acid transporters, and increase the phosphorylation of glycolytic enzymes, which in turn increases glycolytic flux and therefore favors anabolism [33,39]. Furthermore, mTORC1 promotes protein translation by 4EBP and p70S6K phosphorylation [40]. Inhibition of mTORC1 (e.g., with rapamycin) impairs glycolysis upon T cell activation and induces T cell anergy [39].

Adenosine Monophosphate (AMP)-activated protein kinase (AMPK) is a metabolic energy sensor that is another master regulator of T cell activation (Figure 2). In contrast to mTORC1, AMPK drives primarily catabolic metabolic programming in order to restore energy supply in response to stress-induced deprivation [41]. As such, its activation depends on the cellular AMP/ATP ratio, and it promotes mitochondrial ATP synthesis while inhibiting energy burning mTORC1-dependent anabolic pathways [41,42]. AMPK activation is important for memory T cell differentiation [43] that is consistent with a predominant mitochondrial network of these cells. It has also been shown to be important for effector T cell development and metabolic flexibility in response to nutrient deprivation in vivo [44]. AMPK-deficient T cells have a high level of mTORC1 at the basal state, high rate of glycolysis, and a decreased ability to respond to metabolic stress and switch toward catabolic metabolism [42].

**Figure 3:**
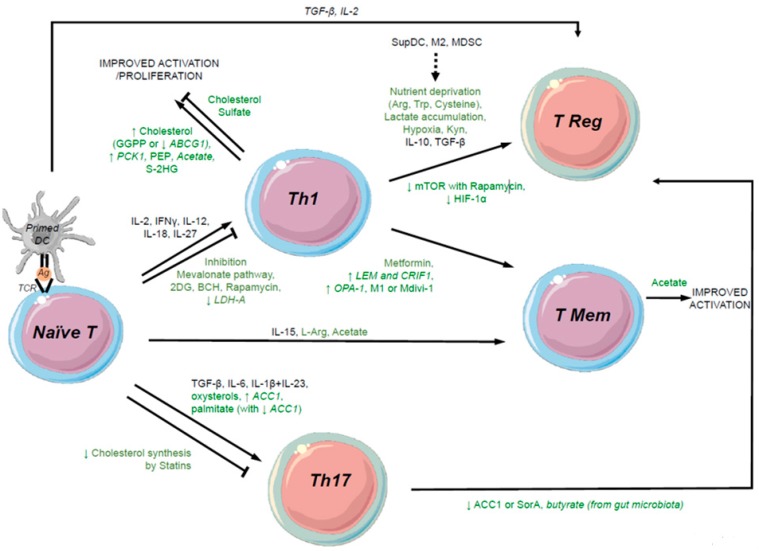
Regulation of metabolic rewiring upon T cell activation. Soluble immune markers that regulate the phenotype of naive T cells, Th1, Th17, TRegs, or T cell memory are indicated in black (cytokines, growth factors), and those related to cell metabolism (macromolecules, metabolites, pharmacological agents) are indicated in green. Green arrows indicate the modulation of metabolic gene expression through overexpression or gene silencing. As described in this figure, the use of various metabolites or the modulation of metabolic gene expression within T cells summarized here (in green) can lead to skew the phenotype and polarization of immune cells, and thereby modulating their immune functions. For example, the treatment of Naïve T cells with the metabolite 2DG or the down-regulation of LDH-A gene expression in these cells can impair their activation and polarization into Th1 phenotype. In addition, the treatment of Th1 cells with metformin or the upregulation of LEM gene expression can improve differenciation into memory T cells. 2DG, 2-deoxy-D-glucose; mTOR, mammalian target of rapamycin; GGPP, geranylgeranyl diphosphate; ABCG1, ATP binding cassette subfamily G member 1; PCK1, phosphoenolpyruvate carboxykinase 1; PEP, phosphoenolpyruvate; S-2HG, S-2-hydroxyglutarate; LDH-A, lactate dehydrogenase A; ACC1, acetyl-CoA carboxylase 1; L-Arg, l-arginine; Trp, tryptophane; CRIF1, CR6-interacting factor 1; OPA-1, optic atrophy protein 1; SorA, soraphen polyketide synthase A; Kyn, Kynurenine; TCR, T cell receptor; Ag, antigen.

HIF-1α is also crucially involved in metabolic reprogramming, enabling T cell adaptation to hypoxic conditions (such as in tissues) (Figure 2). Stabilization of HIF-1α increases glucose uptake and enables the metabolic shift from OXPHOS to aerobic glycolysis [19,20]. HIF-1α expression depends on mTORC1 activity to sustain glycolytic metabolism, and leads to the regulation of the transcriptional programming required for T cell proliferation. Deletion of its negative regulator, von Hippel-Lindau (VHL), enhances T cell glycolysis and effector responses [45]. HIF-1α also induces the accumulation of the metabolite S-2-hydroxyglutarate (S-2HG), which in turn mediates histone and DNA demethylation in activated T cells to modulate the expression of various immune markers [46]. Ex vivo supplementation of this metabolite S-2HG to CD8+ T cells improved proliferation, persistence, and antitumor function after adoptive transfer (Figure 3).

#### 2.3.3. Metabolic Switch and T Cell Phenotypes

Specific metabolic programming has been associated with various T cell phenotypes. Th1, Th2, and Th17 cells use glycolysis via mTORC1 signaling, whereas Regulatory T cells (TRegs) engage mostly FAO [16] (Figure 2). Thus, suppression of mTOR or HIF-1α expression (with rapamycin or genetic deletion) leads to the generation of TRegs, even in cell culture media supplemented with Th17-polarizing cytokines in vitro [47,48]. Th17 cells rely on mTOR- and HIF-1α-dependent glycolysis; blocking glycolysis with 2 deoxy-d-glucose (2DG) in HIF-deficient mice leads to the inhibition of Th17 differentiation [49]. In mice models, TRegs have shown high levels of AMPK and stimulation of AMPK leads to decreased GLUT-1 expression [16]. Blocking glycolysis leads to the inhibition of Th17 differentiation and increased TReg generation [16,50]. Signaling through mTORC1 leads to the activation of quiescent CD4+ T cells into Th1 cells, whereas activation of mTORC2 differentiates CD4+ T cells into the Th2 phenotype [51,52]. Consistent with this, lactate dehydrogenase A (LDH-A) which supports aerobic glycolysis in Th1 polarization, is induced in T cell activation, and promotes Interferon γ (IFNγ) expression by maintaining high concentrations of acetyl-CoA, which enhances histone acetylation and transcription of *Ifng* [53].

In addition to LDH-A, stabilization of IFNγ mRNA is under the control of GlycerAldehyde-3-Phosphate DeHydrogenase (GAPDH) expression, another glycolytic enzyme that binds to AU-rich elements in 3’UTR of IFNγ mRNA when the enzyme is not engaged at a high glycolytic rate [26]. Further investigation of the role of glycolysis in Th1 polarization by Ho et al. [54] has shown that glycolytic metabolite PhosphoEnolPyruvate (PEP) sustains Ca2+ and NFAT signaling involved in IFNγ production. PEP supplementation or overexpression of PhosphoEnolPyruvate CarboxyKinase 1 (PEPCK1) in CD4+ T cells boosted IFNγ production and antitumor function in a melanoma mouse model (Figure 3).

A study that examined the proliferation and survival of activated CD4+ T cells (TCR/CD28 stimulation) using mass spectrometry to quantify protein dynamics revealed rapid remodeling of the mitochondrial proteome with a distinct metabolic signature of one-carbon metabolism [55]. Serine, which accumulates from an increased glycolytic rate, fed the purine and thymidine synthesis to enable T cell proliferation and survival, and gene silencing of mitochondrial serine hydroxymethyltransferase 2 (SHTM2) reduced antigen-specific T cell abundance in vivo in mice and lowered production of inflammatory cytokines IL-17 and IL-6, but not IFNγ or Tumor Necrosis Factor α (TNFα). Hence, mitochondrial function via one-carbon metabolism is important for T cell proliferation in addition to glycolysis for IFNγ production, and the importance of this nucleotide metabolism is also emphasized in acquiring the innate immune memory phenotype of macrophages after Toll-Like Receptor (TLR) stimulation [56]. For TRegs, differences of metabolic requirements using unbiaised proteomics were observed between in vitro cultured cells (both glycolysis and FAO) and freshly-isolated ex vivo cells (highly glycolytic) [57].

### 2.4. Metabolic Switch in Memory T Cells

#### 2.4.1. Metabolic Reprogramming

After activation, the effector T cell population contracts and the majority of cells undergo apoptosis. A small number of activated T cells persist to become memory T cells and during this transition these T cells shift their metabolism to catabolism to support quiescence and long-term persistence. AMPK plays an important role in memory T cell differentiation (Figure 2). In these T cells, the ratio of AMP to ATP increases, leading to the activation of AMPK that promotes FAO [30] to supply mitochondria to TCA cycle intermediates necessary for potent ATP synthesis. Consistently, metformin, which is known to indirectly activate AMPK, enhances the differentiation of memory CD8+ T cells and decreases differentiation of effector T cells [43,58] (Figure 3). As described above, AMPK activity inhibits mTOR and pharmacological inhibition of mTOR enhances memory differentiation as well [42,44,58].

#### 2.4.2. Antigen Recall

FAO is necessary for CD8+ T cells to differentiate into the memory phenotype, but also for their long-term persistence and reactivation after antigen recall [58]. After novel antigen stimulation, memory T cells undergo more rapid differentiation [59] that is made possible by a larger mitochondrial mass (consistent with AMPK activity) and higher spare respiratory capacity (SRC) than naïve or effector T cells. This confers a bioenergetic advantage because mitochondrial SRC increases survival, and FAO enables long-term persistence [10,18]. An early and rapidly increased glycolytic flux in response to TCR/CD28 stimulation was also demonstrated for rapid IFNγ production by effector memory T cells. Such early glycolysis is mediated by CD28-induced Akt and mTORC2 [60] and can feed the mitochondrial TCA cycle with pyruvate to boost mitochondrial oxidative metabolism. Consistent with this mechanism, systemic acetate, which accumulates in response to stress (including bacterial infection), was shown to increase acetyl-CoA levels in memory T cells that in turn mediates GAPDH acetylation to increase enzyme activity, thereby improving rapid IFNγ production [61] (Figure 3). This result is consistent with a study performed by Peng et al. (described above) [53] that established that an increased rate of acetyl-CoA production boosted IFNγ production through epigenetic modifications, and mechanisms that involve acetyl-CoA-induced GAPDH acetylation and histone acetylation may exist simultaneously. Memory T cells are thus metabolically primed to antigen recalls.

Importantly, memory T cells (especially central memory T cells) that infiltrate tumors are involved in efficient and long-lasting antitumor immune response in vivo in various mouse models [62]. These T cells might be more resistant in vivo to the immunosuppressive microenvironment of solid tumors potentially due to the mitochondrial oxidative metabolism that sustains this phenotype and confers bioenergetic advantages [31]. Consistent with this, enforced expression of Peroxisome proliferator-activated receptor Gamma Coactivator 1α (PGC-1α) or OPtic Atrophy type 1 (OPA-1) that increases mitochondrial mass or fused mitochondria in T cells improves antitumor immune function [63,64] (Figure 3). Amino acid metabolism also appears to be important in the central memory phenotype of T cells since high intracellular l-arginine was sufficient to shift glycolysis toward mitochondrial OXPHOS in memory T cells, leading to increased survival and antitumor function [65] (Figure 3). Transcriptional regulators BAZ1B, PSIP1, and TSN appear to be involved in l-Arginine sensing.

Finally, ablation of MCJ (repressor of the mitochondrial respiratory chain) enhances protective activity of memory CD8+ T cells against influenza virus [66]. Therefore, glycolysis and mitochondrial OXPHOS appear to be both important in memory T cells for IFNγ production and persistency, respectively. Based on the hypoxic environment of tissues and tumor microenvironment where memory T cells can reside, one may speculate that the mitochondrial-dependent metabolic advantage is not due to improve respiratory capacity in vivo but rather to the combination of molecular events described above including the accumulation of acetyl-CoA for epigenetic modifications.

### 2.5. The Metabolism of Regulatory T Cells

TReg cell metabolism relies on glycolysis-driven lipogenesis with high lipid oxidation rates [16], with a crucial role of the mevalonate pathway [67] (Figure 2). Furthermore, TRegs, such as effector T cells, are also highly glycolytic, with increased glucose uptake [54] but are less glutaminolytic [68]. It was shown that Glycolysis has a crucial role in the expression of the transcription factor Foxp3 and therefore subsequent TReg immunosuppressive functions. At the molecular level, TRegs express high level of AMPK expression and activity, and low levels of mTOR activation through Phosphatase and Tensin Homolog (PTEN) [69].

## 3. Metabolic Network in Myeloid Cells

Myeloid cells play an important role in homeostasis by stimulating lymphocytes to respond to pathogens [70], eliminating foreign substances, and phagocytizing dying cells. Myeloid cells are considered mediators of innate immune responses to microbial components and even cell stress and tissue damage through the expression of various pattern recognition receptors that sense danger signals and initiate the appropriate immune response. Immature myeloid cells are generated in bone marrow and differentiate according to environment signals into granulocytes, macrophages, mast cells, or dendritic cells (DCs). Once generated, myeloid cells migrate from the bone marrow to tissues by integrating chemokine signaling. In diseased conditions such as cancer, these cells are skewed toward an immunosuppressive phenotype, with the generation of myeloid-derived suppressor cells (MDSCs) and M2-like tumor-associated macrophages (TAM) [71]. This results in a very plastic myeloid compartment dependent on microenvironmental cues, such as cytokines, released by tumor cells and other immune cells.

### 3.1. Metabolic Switch in Macrophages Upon Polarization

#### 3.1.1. Macrophage Differentiation/Subtypes

After their generation in bone marrow, monocytes are released into the blood circulation. They are rapidly directed to various tissues where they remain as immature monocyte-derived-macrophages, acting as immune sentinels. Macrophages differ from DCs, which also derivate from monocytes, by a different expression profile of surface markers. Several subpopulations of macrophages exist based on tissues where they reside, and various subsets of functional macrophages are characterized according to the environmental signals they integrate [72,73]. M1 polarized macrophages play a role in host defense against pathogens through the secretion of inflammatory cytokines (IL-12, IL-6, or IL-1) and reactive nitrogen species (RNS) or reactive oxygen species (ROS) [74]. They are also able to inhibit immunosuppressive cells and amplify Th1 responses [73]. Immature macrophages can also differentiate into M2 macrophages after stimulation with IL-4, IL-10, or glucocorticoids, which have an anti-inflammatory effect through the reduced secretion of inflammatory cytokines, the increased secretion of anti-inflammatory cytokines such as IL-10, and others factors that favor tumor progression and tissue remodeling. TAMs are plastic in response to signals encountered within the tumor microenvironment, as they can reverse their immunosuppressive phenotype under the influence of IFN-γ and produce inflammatory cytokines in vitro [75].

Macrophages are a major component of immune infiltrate in solid tumors and play an important role in tumor phenotype [76]. Through their secretion of inflammatory molecules, they participate in tumorigenesis, increasing inflammatory processes and favoring genomic instability. Furthermore, M2 macrophages inhibit the antitumor immune response via the production of anti-inflammatory cytokines, such as IL-10 or Transforming Growth Factor β (TGF-β), induce tissue remodeling with the secretion of metalloproteinases, and have a pro-angiogenic role through molecules such as Vascular Endothelial Growth Factor (VEGF). A recent study examined tumors that were infiltrated by M1 and M2 macrophages with functional plasticity, in which macrophage metabolism was modulated over time, leading to various macrophage phenotypes [76]. At early stages of cancer development, macrophages harbor a M1 phenotype with increased glycolysis, favoring tumorigenesis through pro-inflammatory functions. At later stages, macrophages likely switch to the M2 phenotype, leading to immunosuppressive functions. This switch is likely favored by tumor-infiltrating Th2 lymphocytes and metabolites such as lactic acid [77,78,79].

#### 3.1.2. Metabolic Networks in M1 and M2 Macrophages

**Figure 4:**
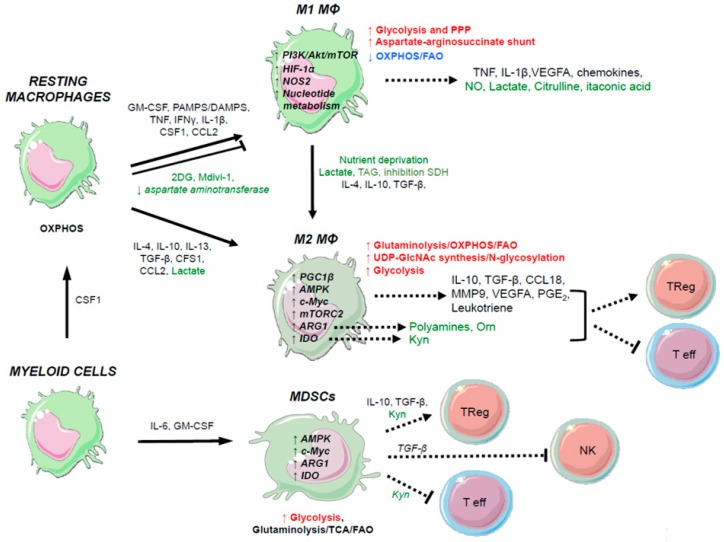
Metabolic rewiring following activation of macrophages. Metabolic requirements are indicated for the differentiation of resting macrophages into M1, M2, or MDSCs. The primary metabolic pathways for each immune phenotype that are upregulated (red), downregulated (blue), or unchanged (black) are indicated. The primary metabolic markers involved in these pathways are indicated in bold black (metabolic enzymes or transcription factors) within immune cells depicted. Soluble immune markers that regulate these differentiation states are indicated in black (cytokines, growth factors, pathogens, danger signals), and those related to cell metabolism (macromolecules, metabolites, pharmacological agents) are indicated in green. Green arrows indicate the modulation of metabolic gene expression through overexpression or gene silencing. As described in this figure, the use of various metabolites or the modulation of metabolic gene expression within macrophages summarized here (in green) can lead to skew the phenotype and polarization of immune cells, and thereby modulating their immune functions. For example, the treatment of resting macrophages by the metabolite 2DG or the down-regulation of aspartate aminotransferase gene expression, can impair their activation into M1 macrophages. 2DG, 2-deoxy-d-glucose; PI3K, phosphoinositide 3-kinase; Akt, protein kinase B; mTOR, mammalian target of rapamycin; HIF-1α, hypoxia-inducible factor 1-alpha; NOS2, nitric oxide synthase 2; AMPK, AMP-activated protein kinase; ARG1, arginase 1; IDO1, indoleamine 2,3-dioxygenase 1; Orn, ornithine; Kyn, kynurenine; TCA, tricarboxylic acid cycle.

As described in Figure 4, M1 and M2 macrophages are characterized by distinct metabolic signatures as evidenced by complementary “omics” approaches underlying their different immune functions. When M1 macrophages are activated by IFN-γ and lipopolysaccharides (LPS), a change to increased glycolytic flux occurs, similarly to activated DCs (Figure 4). This metabolic shift is controlled by the Akt/mTOR/HIF-1α pathway, and leads to a break in the mitochondrial TCA cycle, thereby accumulating intermediates and leading in turn to the expression of IL-1β [72] and TNF cytokines [80]. Among various TCA cycle metabolites, succinate appears to play a crucial role in driving the inflammatory programming of macrophages while down-regulating anti-inflammatory genes; succinate dehydrogenase (SDH) activity triggering mtROS production appears to be required to mediate this transcriptional programming [81]. In contrast, when M2 macrophages are activated by IL-4 and IL-13, they experience a switch to OXPHOS and FAO as well as glycolysis [82]. Increase of glucose metabolism in IL-4-treated macrophages depends on mTORC2 in a pathway that involves PI3K and AkT. Both mTORC2 and Signal Transducer and Activator of Transcription 6 (Stat6) are required to induce the transcription factor Interferon Regulatory Factor 4 (IRF4), leading to increased glucose utilization and promotion of M2 polarization. Loss of mTORC2 in macrophages using Rictor −/− mice but not Raptor−/− mice suppresses tumor growth and protective immunity to the nematode *H. polygyrus*. c-Myc is also a main transcription factor involved in M2 polarization [83]. Indeed, c-Myc supports the metabolic switch to aerobic glycolysis, upregulates glutaminolysis to feed the mitochondrial TCA cycle, and favors fatty-acid synthesis [29].

HIF-1α is one of the main transcription factor that supports the metabolic features of M1 macrophages [84]. Data have shown that p53 is also involved in this alternative polarization, as well as STAT1 and Nuclear Factor-Kappa B (NF-κB) [85]. Under hypoxic conditions, M1 macrophages express HIF-1α, which induces the expression of glycolytic enzymes to favor the shift to glycolysis, induces the production of reactive nitrogen intermediates, and reduces TCA cycle activity and mitochondrial respiration, in turn increasing ROS production for host defense [86]. At early stages of cancer development, macrophages can induce genetic instability and a malignant phenotype by favoring glycolysis. In mouse models, genetic knockout of HIF-1α leads to a diminution of ATP levels in macrophages, and an impairment in migration, invasion, and aggregation processes. These mice had reduced phorbol-myristate acetate (PMA)-induced inflammation, indicating that HIF-1α is involved in the maturation of pro-inflammatory macrophages, consistent with its stabilization by succinate in LPS-treated macrophages [81]. Mitochondrial OXPHOS is required for the maturation of M2 macrophages, with STAT6 and Peroxisome proliferator-activated receptor Gamma Coactivator 1 β (PGC-1β) as the main factors involved. PGC-1β is an important transcriptional regulator of OXPHOS genes that is driven by STAT6 [87] and its knockdown impairs activation of these cells in vitro. In mouse models, expression of PGC-1β leads to a decreased M1 maturation and activation [87]. The accumulation of lactic acid in macrophages (through increased glycolysis) induces an immunosuppressive phenotype for macrophages through the increased expression of arginase-1, VEGFA, and M2 markers [77]. Macrophages also use another important metabolic pathway, glutaminolysis. Macrophages express high levels of glutaminase because glutamine is essential for several macrophage functions (e.g., cytokine production, antigen presentation, and phagocytosis) [7]. M2 macrophages upregulate several genes relevant to the glutamine pathway [77,88] along with UDP-GlcNAc-associated modules [88]. M2 macrophages also metabolize fatty-acids by upregulating fatty-acid uptake and increased FAO [89].

There are distinct patterns of amino acids use, with M1 cells using arginine as a substrate for inducible nitric oxide synthase (iNOS), whereas M2 cells use arginine as a substrate of arginase-1, which produces polyamines involved in cell proliferation and tissue remodeling to favor tumor progression [74]. A system approach identified that M1 macrophages undergo a metabolic break at the mitochondrial isocitrate dehydrogenase (*Idh*) gene, and that an aspartate-arginosuccinate shunt can compensate for this break [88]. This shunt links the TCA and urea cycles through the exchange of fumarate and malate intermediates, through a process called the “Krebs bicycle”. Aspartate, produced by transamination of glutamate and oxaloacetate in mitochondria, can be released into the cytosol where it acts as a nitrogen donor in the urea cycle after its transformation in argininosuccinate. This reaction is called the aspartate-argininosuccinate shunt, and the balance between these two cycles is regulated by the transport of intermediates between the cytosol and mitochondria.

The role of this shunt was further confirmed in a subsequent study that allowed the accumulation of fumarate and in turn an inflammatory epigenetic reprogramming through histone-acetyl transferases and methylation; these modifications ultimately provided transcriptional production of inflammatory cytokines TNFα and IL-6 [90]. In addition to the study on dectin-1, stimulation of M1 macrophages (a similar triggering of epigenetic modifications) were also observed in response to LPS and subsequent TLR signaling [56]. These modifications consistently regulate various metabolic pathways at the transcriptional level, including acylglycerol degradation, lipid metabolism, and pyrimidine ribonucleotide biosynthesis [56]. These studies demonstrate that epigenetic-induced metabolic rewiring is involved in the acquisition of the M1 inflammatory phenotype following TLR or dectin-1 stimulation, and illustrates the crucial role of metabolic reprogramming in establishing the trained immunity (long-term innate immune memory) as described in Netea et al. [91].

### 3.2. The Metabolism of Dendritic Cells

#### 3.2.1. Function

These cells are traditionally described as the frontier of innate and adaptive immunity. They induce primary immune responses because of their antigen-presentation function, and they also have an important role in priming T lymphocytes. Activation and maturation of immature DCs depends on their interaction with pathogen-associated molecular pattern (PAMPs) or damage-associated molecular pattern (DAMP) molecules. This leads to the upregulation of antigen-presenting molecules, cytokines, and chemokine receptors. Once activated, mature DCs migrate to lymphoid organs where they are efficient in their ability to induce antigen-specific T cell activation.

#### 3.2.2. Metabolic Network in DCs

**Figure 5:**
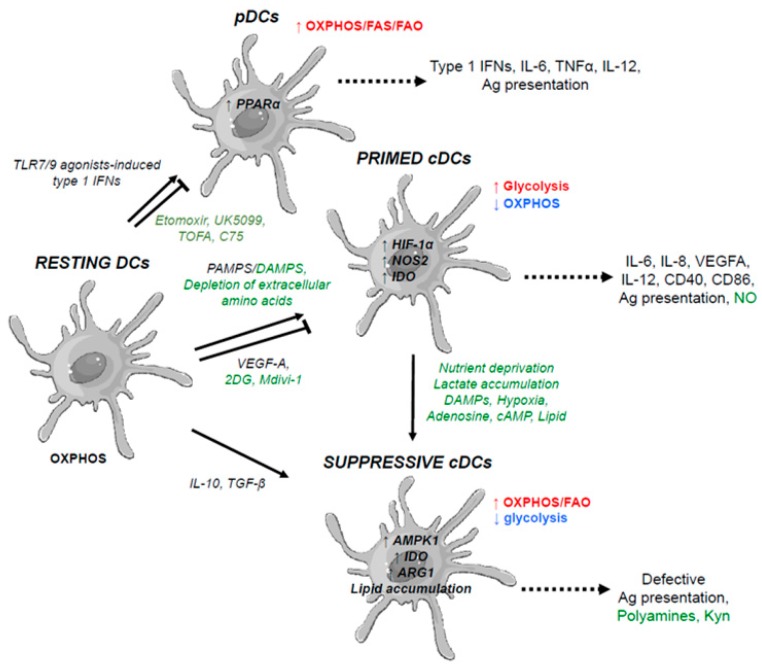
Metabolic rewiring following activation of dendritic cells. Metabolic requirements are indicated for the differentiation of resting dendritic cells (DCs) into plasmacytoid DCs, primed DCs, or suppressive DCs. The primary metabolic pathways for each immune phenotype that are upregulated (red), downregulated (blue), or unchanged (black) are indicated. The primary metabolic markers involved in these pathways are indicated in bold black (metabolic enzymes or transcription factors) within immune cells depicted. Soluble immune markers that regulate these differentiation states are indicated in black (cytokines, growth factors, pathogens, danger signals), and those related to cell metabolism (macromolecules, metabolites, pharmacological agents) are indicated in green. Green arrows indicate the modulation of metabolic gene expression through overexpression or gene silencing. As described in this figure, the use of various metabolites or the modulation of metabolic gene expression within DCs summarized here (in green) can lead to skew the phenotype and polarization of immune cells, and thereby modulating their immune functions. 2DG, 2-deoxy-d-glucose; HIF-1α, hypoxia-inducible factor 1-alpha; NOS2, nitric oxide synthase 2; AMPK, AMP-activated protein kinase; ARG1, arginase 1; IDO1, indoleamine 2,3-dioxygenase 1; Orn, ornithine; Kyn, kynurenine; TCA, tricarboxylic acid cycle; NO, nitric oxide; TLRs, toll-like receptors; IFNs, interferons; PPARα, peroxisome proliferator-activated receptor alpha.

Quiescent DCs primarily use OXPHOS to produce energy and FAO (Figure 5). After pathogen stimulation, there is a shift to glycolysis with decreased OXPHOS [92]. It appears that this shift occurs very early after TLR-dependent activation of DCs. Indeed, inhibition of glycolysis via 2DG leads to impairment of the expression of activation markers [92]. In DCs, the switch to Warburg metabolism is not necessary for proliferation (unlike in tumor cells), but supplies new cellular functions and biosynthetic activities at early phases of activation. Furthermore, this metabolic shift produces ATP, which increases their survival at later phases of activation (by maintaining mitochondrial membrane potential and inhibiting cytochrome c release). This is favored by the activation of HIF-1α and PI3K/Akt pathways, leading to increased uptake of glucose (via the glucose transporter GLUT-1), increased activity of hexokinase and phosphofructokinase (both are glycolysis enzymes), increased production of lactate, and decreased mitochondria oxidative metabolism and β-oxidation [92]. Knockdown of HIF-1α in mouse models leads to reduced glucose uptake, inhibition of their maturation, and impaired capacity to prime T cells [93]. Additionally, diminution of oxidative metabolism is favored by iNOS, which inhibits mitochondrial reoxidation of NADH by the production of NO, and activation of PI3K/Akt pathway, which inhibits AMPK (a master regulator of OXPHOS).

Under hypoxic conditions, the activation of HIF-1α induces the expression of glycolytic genes and increases glycolysis. This induces an increase of DC maturation in mouse models [93], however, hypoxia can limit their migration and differentiation in human models [94]. Furthermore, hypoxia, through adenosine and lactate accumulation, inhibits their activation [95]. These adenosine-induced DCs express several tolerogenic molecules (such as TGF-β, IL-6, IL-10, and proangiogenic molecules like VEGFA) [96]. Adenosine also induces the expression of AMPK, thereby inhibiting glycolysis and favoring the shift toward mitochondrial OXPHOS, and reduces activation.

## 4. The Emergence of Specific Metabolic Targets to Modulate Immune Cell Effector Functions

As discussed above, efforts have been made to thoroughly characterize the intricate metabolic networks engaged in immune cells, both in response to in vitro stimuli (including antigens or PAMPs) or ex vivo in various physiological settings. As a result, metabolic peculiarities have been successfully delineated for some immune cell types, though limitations of the ex vivo experimental procedure prevent it from completely reflecting the complexity of the in vivo microenvironment. In addition to mapping metabolic networks of immune cells in a given phenotype, crucial insights have been recently gleamed, establishing that manipulation of metabolic enzymes is sufficient to modulate the phenotype of immune cells. Importantly, this metabolic manipulation of immune phenotype has been demonstrated for various immune cell types, including innate macrophages and adaptive T cells (CD4+ or CD8+). Such knowledge is of particular interest for therapeutic intervention in inflammatory diseases and cancer, where it may be possible to metabolically fine-tune immune effector function from harmful toward protective based on disease context, or to even improve persistency of exhausted tumor infiltrating cytotoxic T cells in cancer. Even though this goal cannot be reached yet in vivo due to the strong heterogeneity of immune cell populations and phenotypes, the range of metabolic targets that control the fate of immune effector function is emerging.

In macrophages, mitochondrial SDH appears to play a major role in controlling the switch between pro- and anti-inflammatory phenotypes [81]. In a study performed by Mills et al., the authors demonstrated that SDH activity in response to LPS is essential for producing pro-inflammatory cytokines. Specifically, succinate-induced SDH activity maintains a high mitochondrial membrane potential (ΔΨm) to generate mtROS that in turn stabilizes HIF-1α to drive pro-IL-1β gene expression. The decrease of ΔΨm via the respiratory chain uncoupler CCCP or Complex I inhibitor rotenone blocks this process. Expression of alternative oxidase (AOX) in bone marrow-derived macrophages (BMDMs) impaired succinate-induced ROS production and associated increase of IL-1β production and HIF-1α. In addition, inhibition of SDH activity by dimethyl malonate (DMM) also impaired production of pro-inflammatory cytokines. Inhibition of SDH activity by DMM represses transcriptional programming of genes associated with inflammation including IL-1β, HIF-1α target genes, and genes involved in fatty-acid synthesis. Conversely, succinate boosted inflammatory genes while decreasing anti-inflammatory gene expression (including IL-10 and IL-1RA). The underlying mechanisms that explain this co-regulation of transcriptional programming needs to be further explored and may be linked to epigenetic modifications through the succinate-mediated inhibition of HIF-1α hydroxylation and DNA methylation (inhibition of PHDs, jmj-KDMs, and TET enzymes) [81]. Specific inhibitors of SDH might be useful for the treatment of inflammatory and autoimmune diseases by dampening the inflammatory phenotype of activated macrophages, and potentially other innate and autoreactive T cells.

## 5. Conclusions

Remarkable achievements have been recently reached in reconstructing highly complex and interconnected metabolic networks in immune cells, and by applying this approach to various immune cell types from both the innate and adaptive immune system. Such outcomes have become possible by implementing complementary technologies to map metabolic changes as accurately as possible. Such approaches combine the expression of metabolic genes and proteins with high-throughput “omics” technologies (transcriptomics, proteomics) at a given steady state of immune activation with fluxomics (metabolomics, isotope tracing) to identify the levels of metabolite intermediates and their biochemical origin. In depth data analyses of integrated “omics” have successfully led to the precise mapping of metabolic circuity in response to various innate or adaptive immune stimuli, clearly establishing that metabolic changes not only occur in response to these stimuli, but more importantly, that a deep metabolic reprogramming is indispensable to skew immune cell functions toward particular phenotypes. Some examples discussed here have recently elucidated the metabolic-induced epigenetic modifications that underlie these modulations of immune function.

Most of these techniques have been applied in in vitro or ex vivo experimental settings, and in some cases to particular enriched immune cell populations. In these in vitro approaches, inconsistencies can easily arise between studies simply due to minor differences in nutrient compositions of cell culture media (including levels of glucose, glutamine, pyruvate, oxygen pressure, and amino acids). These nutrient levels can vary in in vivo tissues and shape the immune cell metabolism in various ways; this might depend on a number of parameters dependent on the tissue itself and interactions between immune cells and their microenvironment. The current limitation now resides in the assessment of immune metabolic signatures in this complex in vivo tissue microenvironment. In the need to translate metabolic studies of immune cells into in vivo settings, a promising approach in translating immune cells metabolic studies into the in vivo environment is cellular-scale tissue microdissection coupled to proteomics analysis. Such an approach can specifically target immune cells of interest within tissue sections and offers the advantage of addressing protein expression of a large panel of metabolic markers to reconstitute circuity. It may also prove useful for translating immune cell metabolism into the clinic through the identification of biomarkers and therapeutic metabolic targets in a broad spectrum of complex human diseases as diverse as metabolic syndrome, inflammatory diseases, or cancer.

The full clinical impact of immune cell metabolism is still undetermined in various contexts, and some positive effects of metabolic drugs (metformin, rapamycin, simvastatin, methotrexate) prescribed for inflammatory diseases might actually contribute to the modulation of immune cell function to some extent. As eagerly awaited clinical trials have begun testing metabolic molecules to prevent and treat various disease indications, the rapidly expanding clinical impact of metabolic targeting for a broad spectrum of immune-mediated illnesses will be further elucidated. Determining the Achilles’ heel of the specific immune cell population for therapeutic intervention remains outstanding. Progress has been already made to establish that adoptive transfer of T cells pretreated with metabolites or isolated from genetically modified metabolic enzymes can improve memory T cell persistence, which correlates with antitumor immune function in mice models. This has translational impact on the generation of persistent memory Chimeric Antigen Receptor (CAR) T cells through the expression of 4-1BB-induced metabolic reprogramming, which increases mitochondrial biogenesis [97], or the generation of more potent and persistent T cells ex vivo for subsequent adoptive transfer.
